# Comparison of screening methods for high-throughput determination of oil yields in micro-algal biofuel strains

**DOI:** 10.1007/s10811-012-9947-5

**Published:** 2012-12-14

**Authors:** Stephen P. Slocombe, QianYi Zhang, Kenneth D. Black, John G. Day, Michele S. Stanley

**Affiliations:** Scottish Association for Marine Science, Scottish Marine Institute, Oban, Argyll PA37 1QA UK

**Keywords:** Intelligent screening, In situ transesterification, Lipids, Biodiesel, Biofuel, *Chlorella*

## Abstract

The phenotypic and phylogenetic diversity of micro-algae capable of accumulating triacylglycerols provides a challenge for the accurate determination of biotechnological potential. High-yielding strains are needed to improve economic viability and their compositional information is required for optimizing biodiesel properties. To facilitate a high-throughput screening programme, a very rapid direct-derivatization procedure capable of extracting lyophilized material for GC analysis was compared with a scaled-down Folch-based method. This was carried out on ten micro-algal strains from 6 phyla where the more rapid direct-derivatization approach was found to provide a more reliable measure of yield. The modified Folch-based procedure was found to substantially underestimate oil yield in one *Chlorella* species (*P* < 0.01). In terms of fatty acid composition however, the Folch procedure proved to be slightly better in recovering polyunsaturated fatty acids, in six out of the ten strains. Therefore, direct-derivatization is recommended for rapid determination of yields in screening approaches but can provide slightly less compositional accuracy than solvent-based extraction methods.

## Introduction

Many micro-algae species have the ability to accumulate oil to high levels in the form of non-polar glycerolipids such as triacylglycerol (TAG) (Day et al. 2012; Hu et al. 2008). This, along with high biomass yields, as well as their capacity to photosynthesise efficiently, has attracted much recent interest in algae as a feedstock for renewable energy fuels and other biotechnological purposes. The taxonomic and ecological diversity of micro-algae provides a resource that can be explored using high-throughput screening approaches to identify those with the most potential. For large-scale production, most workers are focussing on strains able to grow in seawater and/or under brackish conditions to avoid competition for agricultural freshwater supplies (Day et al. 2012; Radakovits et al. 2012). Rapid and accurate methods of evaluating oil content and composition are required for high-throughput screening strategies. Fatty acid composition affects key biodiesel characteristics such as cetane number, oxidative stability and cold flow (Ramos et al. 2009). Therefore, strain selection is essential in order to drive down economic costs and optimize biodiesel properties (Day et al. 2012).

Surveys using fluorescent stains such as Nile Red in combination with flow cytometry, or plate assays have previously been undertaken (Chen et al. 2009; Sheehan et al. 1998). Nevertheless, when comparing oil content between species, variation in dye absorption efficiency, cell size and calibration issues must be taken into consideration (Chen et al. 2009). Compositional information is also required therefore an alternative or complementary approach is rapid lipid quantification via gas chromatographic (GC) analysis of fatty acid methyl esters (FAMES), where GC-flame ionization detector (GC-FID) provides the greatest quantitative accuracy (Christie 1993). Here, the main potential caveats are found in sample processing time and extraction efficiency.

Earlier surveys of micro-algal oil content and composition based on GC analysis have employed solvent extraction of fresh (Dunstan et al. 1992; Volkman et al. 1989) or lyophilized material (Ben-Amotz et al. 1985; Shifrin and Chisholm 1981) followed by derivatization to FAMEs. Most published extraction procedures applied to micro-algae have utilized chloroform–methanol based extraction according to Bligh and Dyer (1959), or Folch et al. (1957). Of these, the Folch procedure has been demonstrated to give better recoveries with high oil content material (Iverson et al. 2001). This implies that the Folch method should be more appropriate for many micro-algae and this was observed by Griffiths et al. (2010) in a methodological comparison on three algal strains. Nevertheless, many other methods employing different solvents have been applied to algae. For instance, some micro-algal procedures extract initially in isopropanol to reduce the possibility of lipase activity (Hodgson et al. 1991; Volkman et al. 1989).

A further consideration in a screening strategy for oil content is that these data are more informative when expressed relative to dry weight (DW) rather than fresh weight (FW). Methods that extract wet biomass, fresh or un-dried material consequently require a separate DW determination step. This adds to the processing time and can introduce errors from FW measurement of the harvested micro-algal material due to liquid carryover, especially at small scale. Therefore, extraction of lyophilized material is more suited to high-throughput screens, but can often be resistant to lipid extraction (Christie 1993). Consequently, additional steps are frequently incorporated into the solvent-based extraction procedures for dried micro-algal samples. For instance, with *Nannochloropsis*, such procedures have included multiple solvent extractions, or extended sonication (Chiu et al. 2009; Hodgson et al. 1991). Although extraction of fresh micro-algal samples can be faster, incomplete lipid extraction can also occur here, where additional steps to aid extraction may be required for some taxa (e.g. *Nannochloris*) (Volkman et al. 1989).

Direct-derivatization methods offer a rapid alternative for high-throughput screening since extraction and derivatization occur simultaneously (Carrapiso and García 2000). In addition, extraction of dried samples could be facilitated under the thermal conditions associated with derivatization. Most procedures utilize anhydrous methanol with acid or base catalysts. Acid catalysts are most appropriate for total fatty acid screens with a view towards biodiesel production because free fatty acids are also derivatized as well as those esterified to glycerolipids (Christie 1993). In some cases, direct-derivatization methods applied to micro-algae have used boron trifluoride with methanol (Me-BF_3_) using the original method of LePage and Roy (1984) or further developments (Griffiths et al. 2010). These have included extraction of marine *Chlorella* sp., for instance (Hsieh and Wu, 2009). The comparative study by Griffiths et al. (2010) reported that their Me-BF_3_ based method produced slightly higher yields than the Folch method on non-dried samples of *Chlorella vulgaris*, *Nannochloropsis* and *Selenastrum*. Unfortunately, reports indicate that the Me-BF_3_ is prone to artefact generation in FAMES preparation (Carrapiso and García 2000; Christie 1993).

Direct derivatization has also been carried out with 2 % (*v*/*v*) H_2_SO_4_ in methanol on *Nannochloropsis* sp. and *Haematococcus pluvialis* (Recht et al. 2012). Concentrated HCl solution combined with methanol has also been used as methanol/conc. HCl/chloroform (10:1:1, *v*/*v*/*v*) applied to dried thraustrochrytid samples (Lewis et al. 2000). In a similar procedure, a 2:1 (*v*/*v*) chloroform/methanol pre-soak followed by 5 % (*v*/*v*) conc. HCl/methanol was applied to *Nannochloropsis* sp., *Chlorella vulgaris* and *Phaeodactylum tricornutum* (Laurens et al. 2012). Nevertheless, the methanolic-HCl reagent (Me-HCl) is more commonly employed in anhydrous form either as commercial stock or prepared by adding acetyl chloride or HCl gas to methanol (Carrapiso and García 2000; Christie 1993). Use as methanol/acetyl chloride has been reported for the red marine unicellular algae *Porphyridium cruentum*, the diatom *P. tricornutum* (Cohen et al. 1988; Rodríguez-Ruiz et al. 1998) and as dry HCl gas in methanol, for *Pavlova lutheri* and *Chaetoceros muelleri* (Jacobsen et al. 2012). Methods using commercial anhydrous Me-HCl stock have been reported for both fresh (Browse et al. 1986) and dried (Larson and Graham 2001) higher plant materials. The latter method is one of the simplest since derivatization is carried out in the presence of hexane; therefore, FAME recovery requires only the addition of aqueous phase.

Although the direct-derivatization approach is becoming more widely used, a wide variety of methods have been applied to relatively few micro-algal species. Comparative studies that examine a wide range of taxa have not been carried out. Overall, the direct methods that included hexane in the derivatization reaction were the simplest to carry out, requiring only the addition of aqueous phase to recover FAMEs (Larson and Graham, 2001; Rodríguez-Ruiz et al. 1998). The other methods reported for micro-algal or terrestrial plant material all extracted FAMES by adding solvent after derivatization, adding to handling time and some included additional processing steps (Browse et al. 1986; Cohen et al. 1988; Griffiths et al. 2010; Jacobsen et al. 2012; Laurens et al. 2012; Lewis et al. 2000).

Therefore, the aim of this study was to compare one of the most rapid direct-derivatization procedures with a scaled-down modified Folch procedure on a representative set of micro-algal strains from the major phylogenetic groupings. The objective was to select a method suitable for high-throughput screening that was applicable over a broad taxonomic group, produced a minimum of artefacts and was capable of extracting lyophilized micro-algae samples with resilient cell walls.

## Materials and methods

Ten marine micro-algae species from six different phyla were analysed (Table 1). All strains were obtained from the Culture Collection of Algae and Protozoa (CCAP, UK). Starter cultures of 100 mL were incubated under a 12 h/12 h light/dark (L/D) regime at 50–80 μmol photons m^−2^ s^−1^ at 20 °C for 7–10 d, without shaking (Innova 44, New Brunswick Scientific). Cultures were monitored by dual measurement of in vivo chlorophyll fluorescence using a Trilogy® Laboratory Fluorometer (Turner Designs, USA) with Trilogy Module CHL-INVIVO (GUI selection BLUE, #043) and cell turbidity by absorbance at 735 nm (*A*_735_) using a NanoPhotometer™ (Implen, Germany). Starter cultures were used for subculturing once they had reached either an *A*_735_ of 0.34 or an in vivo chlorophyll fluorescence of 10,000 RFU (Relative Fluorescence Units). In the case of *Nannochloropsis oculata* (CCAP 849/1) these values both equated to 1 × 10^7^ cells mL^−1^. Sub-samples were then inoculated at 5 % (*v*/*v*) into 3 × 500 mL aerated Erlenmeyer flasks containing 400 mL F/2 medium (Guillard and Ryther 1962) using artificial seawater at 33.5 g L^−1^ (Instant Ocean, Aquarium Systems, France). Each flask was capped with a ported GL45 connection system (Duran, Germany) enabling sterile filtration of input air (HEPA-VENT, Whatman, UK). Aeration and culture mixing was accomplished by bubbling air at 60 mL min^−1^ through a 4-mm bore silicone tube (Apex, UK) that reached to the base of the flask. Each flask was exposed to 150 ± 10 μmol photons m^−2^ s^−1^ (except for the rhodophyte *Porphyridium purpureum* (CCAP 1380/1A), which was exposed to 50 μmol photons m^−2^ s^−1^) of photosynthetically active radiation (PAR, 400–700 nm). Two types of fluorescent tubes were used (Osram Lumilux Warm White L30W/830, and GE Cool White F30W/33-640) for 16 h/8 h L/D, at 20 °C throughout, in a controlled environment room. Once the cultures reached stationary phase (defined by no further increase in at least one of the dual parameters of turbidity and in vivo chlorophyll fluorescence, with sampling at 1–2-day intervals) they were harvested by centrifugation at 4000×*g* for 15 min. The harvested cells were then flash-frozen in liquid nitrogen, and then freeze-dried for 3 days. Freeze-dried algae were then transferred to individual glass vials, with a Teflon-lined stopper, and stored in the dark, under nitrogen gas at −80 °C.

**Table 1:** Comparison of FAME yields (%DW) for direct-derivatization and Folch methods

Micro-algal strain				Direct		Folch		Yield ratio
Class	Genus	Species	CCAP No.	Mean^a^	SD	Mean^a^	SD	
Chlorophyceae	*Chlorella*	*ovalis*	211/21A	51.0^b^	3.4	34.9^b^	3.9	1.46
	*Chlorella* sp.		211/75	59.8	1.8	58.9	1.8	1.01
	*Dunaliella*	*tertiolecta*	19/27	9.9	2.2	11.1	2.4	0.90
	*Tetraselmis*	*chui*	66/21A	5.3	1.4	5.4	1.8	0.99
	*Tetraselmis* sp.		66/60	12.6	0.3	12.9	0.4	0.98
Bacillariophyceae	*Thalassiosira*	*pseudonana*	1085/12	15.2^b^	0.3	16.2^b^	0.2	0.94
Eustigmatophyceae	*Nannochloropsis*	*oculata*	849/1	21.0	1.2	24.6	2.4	0.85
Cryptophyceae	*Rhinomonas*	*reticulata*	995/2	45.5	1.9	46.3	1.4	0.98
Prymnesiophyceae	*Isochrysis*	*galbana*	927/1	9.1	0.7	9.9	3.2	0.91
Rhodophyceae	*Porphyridium*	*purpureum*	1380/1A	7.6	0.7	8.1	1.0	0.94

^a^Mean of three independent biological replicates, standard deviation (SD) indicated

^b^Significant difference determined by *t* test (*P* < 0.01)

### Direct-derivatization procedure

The rapid small-scale direct-derivatization procedure was carried out essentially according to Larson and Graham (2001). Lyophilized micro-algae material (10 mg) was weighed into 2 mL screw-top vials (Chromacol, UK) and 200 μL hexane containing 0.01 % (*w*/*v*) butylated hydroxytoluene (BHT) (Sigma) was added. This was followed by 10 μL methyl tricosanoate (Larodan, Sweden) internal standard (5 mg mL^−1^ in hexane) and 500 μL anhydrous 1 N methanolic-HCl (Sigma). The vials were flushed with nitrogen and capped with Teflon seals prior to incubation at 85 °C for 2 h. After cooling at room temperature, 250 μL of 0.88 % (*w*/*v*) KCl was added and the upper hexane phase containing FAMES was removed to Teflon-capped tapered vials (Chromacol) for GC analysis, flushing the sample with nitrogen gas before capping. Samples were either run immediately or stored under nitrogen at −80 °C.

### Modified Folch procedure

The extraction procedure was carried out according to Folch et al. (1957) with modifications, and derivatized according to Cook et al. (2000). Lyophilized micro-algal material (25 mg) was ground in a chilled mortar and pestle with 5 mL of chloroform/methanol (2:1, *v*/*v*) with 0.01 % (*w*/*v*) BHT and 10 μL methyl tricosanoate internal standard (5 mg mL^−1^ in hexane). This was followed by the addition of 250 μL purified water (Purelab ULTRA, ELGA Process Water, UK) and more grinding. Further homogenization was carried out using a 15-mL glass Potter’s homogenizer (Wheaton, USA) to achieve a fine suspension. Samples were transferred to 8 mL Pyrex screw-capped tubes (Corning), flushed with nitrogen and incubated for 3 d at 4 °C and then re-extracted with the homogenizer. The solvent extract was filtered through glass-wool using a glass Pasteur pipette and mixed with 25 % vol of an aqueous wash of 0.88 % (*w*/*v*) KCl. This was allowed to partition and the lower solvent phase was transferred to a 25-mL Pyrex screw-capped tube and evaporated under a stream of nitrogen. The resultant lipid extract was derivatized by addition of 1.5 mL toluene (containing 0.01 % (*w*/*v*) BHT) and 3 mL of a fresh stock of 1 % (*v*/*v*) H_2_SO_4_ in methanol and incubated at 100 °C for 2 h, under nitrogen. On cooling, 2 mL of 5 % (*w*/*v*) KCl was added and FAMES partitioned twice into 2 mL hexane/ether (1:1, *v*/*v*) with 0.01 % (*w*/*v*) BHT. To the combined hexane–ether extracts, 2 mL of 2 % (*w*/*v*) NaHCO_3_ was added for acid neutralization, collecting the top solvent layer. This was evaporated under nitrogen and resuspended in hexane (0.01 % *w*/*v* BHT) for GC analysis.

### Gas chromatography

Hexane phases containing FAMES were transferred to Teflon-capped tapered vials (McQuilkin, UK) and 1 μL aliquots were analysed by GC-FID (GC-2014, Shimadzu, Kyoto, Japan). Injections were made into a 30-m, 0.25-mm ID ZB-wax column (Phenomenex, Denmark) using helium as carrier at 1.56 mL min^−1^ with a split ratio of 100:1. The oven was ramped from 160 °C to 240 °C at 4 °C min^−1^ then run isothermally at 240 °C for 10 min. Peak areas were integrated using GC solution software (Shimadzu, Japan) and quantified by reference to the internal standard when expressing as %DW. Peak areas were also converted to moles and expressed as a percentage of the total identified FA (mol%). Defined classes such as total unsaturated FA were expressed as the sum of individual mol% or %DW values corresponding to individual FA. Blank runs were generated from both extraction methods by performing the entire procedures without biological material. Peak identities were ascertained using external standards: 37 FAMES, PUFA2, PUFA3 (Sigma), methyl 9(*Z*), 12(*Z*) Hexadecadienoate (Larodan) and Methyl 7(*Z*) hexadecadienoate (Cambridge Biosciences, UK). Further analysis by GC-MS (Trace GC2000, Thermoquest CE Instruments, USA) was carried out (conditions as above except that He was 1.0 mL min^−1^ with a split ratio 1:50) to establish peak identity based on mass spectra (Xcalibur software, Thermoquest, CE instruments) using DMOX-derivatization of FAMEs samples to establish double-bond position (Fay and Richli 1991).

## Results

### Strain selection and FAMES preparation

Ten marine micro-algae species from six different phyla were selected for the comparison of lipid extraction methods (Table 1). These included *Chlorella* and *Nannochloropsis* strains where extraction issues were expected to arise (Chiu et al. 2009; Doucha and Lívanský 2008). For the comparison, micro-algae cultures were grown in triplicate to stationary phase under controlled environment conditions. Each replicate was harvested and lyophilized then extracted once with a direct-derivatization method and a scaled-down modified Folch procedure.

The first method involved co-incubation of dried material with hexane, internal standard and the derivatization reagent 1 N Me-HCl. This was followed by addition of aqueous buffer and recovery of hexane (Larson and Graham 2001). This was chosen because the anhydrous Me-HCl reagent has widespread use with few reports of artefacts and this particular method is one of the most rapid and requires minimal handling. It was anticipated that exposure to the 1 N Me-HCl reagent at 85 °C would promote extraction, given its corrosive nature. An aqueous KCl wash facilitated separation of FAMEs from polar by-products. Although the incubation period was 2 h, the rest of the process required less than 5 min handling time per sample.

The scaled-down Folch procedure was optimized for dried micro-algae material (Folch et al. 1957). This entailed homogenization in methanol/chloroform (2:1, *v*/*v*) plus a small quantity of water added to improve extraction of lyophilized material. Extraction and purification of lipids was followed by derivatization with methanolic sulphuric acid and an acid neutralization step (Cook et al. 2000). Handling time was 30 min per sample for both the extraction and derivatization steps. Suspension by homogenization proceeded relatively rapidly in some strains such as *Porphyridium*. In the case of marine *Chlorella* strains, more intensive mechanical homogenization was needed for suspension and prolonged incubation at 4 °C was required to remove chlorophyll from the pellet. The difficulty in removing chlorophyll from the marine *Chlorella* strains suggested a general impediment against solvent extraction.

### Oil content estimation

The objective was primarily to compare estimates of oil content (as total FAMEs %DW) and fatty acid composition. Both of the methods, as applied here, were adapted to small-scale, high-throughput use, therefore total recovery was not a prime consideration. Differences in total recovery were obviated though the use of an internal standard in content estimation, so the data presented here depend principally on extractability and actual oil content. Comparisons were carried out on triplicate cultures so error included biological variability and extraction efficiency components. The total FAMEs content (%DW) measurements are compared for the two procedures in Table 1. Micro-algae oil contents at stationary phase ranged from 5–60 %DW, with the highest oil producers being the marine *Chlorella* strains, the cryptophyte *Rhinomonas reticulata* (CCAP 995/2) and *N. oculata* (CCAP 849/1). These data were similar for the two methods except in the case of *Chlorella ovalis* (CCAP 211/21A) where the estimated content was 46 % higher with the direct-derivatization method (*P* < 0.01). In the case of the diatom *Thalassiosira pseudonana* (CCAP 1085/12), oil content was lower with the direct method compared with the Folch method but only by about 5 % (*P* < 0.01). With the other 8 strains, no statistically significant changes were observed in oil content.

### Fatty acid composition

**Fig. 1:**
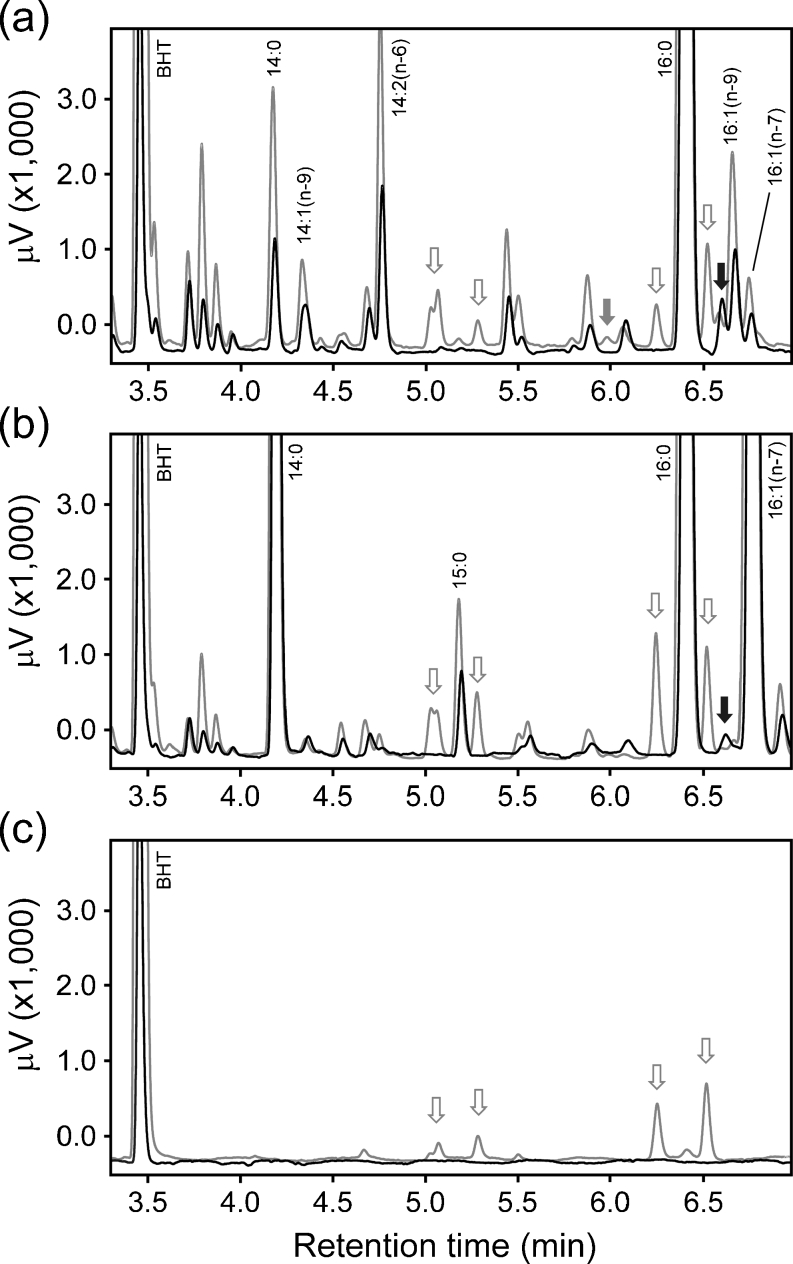
Representative GC-FID traces from **a** *D*. *tertiolecta* CCAP 19/27; **b** *T*. *pseudonana* CCAP 1085/12 and **c** blank runs, focussing on the region where minor non-fatty acid peaks were typically located. Overlaid traces are from direct-derivatization (*black*) and Folch (*grey*) preparations from the same cultures. The anti-oxidant BHT and FAMES peaks (identified according to mass spectra and DMOX-derivatization) are labelled. *Arrows* indicate non-FAMEs peaks that were specific to the Folch (*grey*) or direct-derivatization (*black*) methods; *open arrows* indicate peaks also present in blank runs and *closed arrows* indicate peaks absent from blank runs

Representative traces are shown for two micro-algae species, *Dunaliella tertiolecta* (CCAP 19/27) and *T*. *pseudonana* (Fig. 1). Traces obtained using the two methods are overlaid and all peaks that were identified as fatty acids are indicated. With both methods, most non-fatty acid peaks (identified as such from their mass spectra) eluted between the 14:0 peak and the 16:1 peaks. The prominent non-FA peaks eluting between 3.5–4.0 min were tentatively identified as phytol and its derivatives and were present in all the strains. Several non-FA peaks were specific to the Folch extracts but were also present in blank runs (Fig. 1c). The direct method was found to introduce relatively few artefacts into the analysis and none were present in blank runs. A single minor non-FA peak commonly appeared between 16:0 and 16:1 (*n*−9) that was absent, or less prominent, in Folch extracts, as previously noted with the model terrestrial plant *Arabidopsis* (Browse et al. 1986). A lesser number of solvent handling steps in the direct method probably led to fewer artefacts overall, compared with the Folch method.

Relative fatty acid composition data (mol%) are depicted for five green-algae phyla (Table 2) and for five non-green micro-algae phyla (Table 3) which were obtained using the direct-derivatization and Folch methods. Fatty acids that comprise >1 % total fatty acids (on a mol% basis) in any one of the ten species are shown here. Overall mean relative standard deviation for these quantified peaks was 7.8 %. Significant differences in fatty acid mol% composition between the two extraction methods were of relatively low occurrence for the major fatty acids. This was seen in the two *Chlorella* strains and *D*. *tertiolecta* in Table 2; *T*. *pseudonana*, *N*. *oculata* and *R*. *reticulata* in Table 3. Although the differences amounted to less than ±10 % peak area between the two methods, there was nevertheless a consistent bias towards higher saturated FAs and MUFAs, with a lower estimation of polyunsaturated fatty acid content with the direct method.

**Table 2:** Green micro-algae FAME composition (mol%) at stationary phase comparing the direct and Folch methods

Fatty acid	Chlorophyceae						Prasinophyceae			
	*C*. *ovalis*		*Chlorella* sp.		*D*. *tertiolecta*		*T*. *chui*		*Tetraselmis* sp.	
	CCAP 211/21A		CCAP 211/75		CCAP 19/27		CCAP 66/21A		CCAP 66/60	
	Direct	Folch	Direct	Folch	Direct	Folch	Direct	Folch	Direct	Folch
Saturated fatty acids										
14:0	−^a^	−	−	−	0.9	1.2	1.5	1.8	−	−
16:0	14.5	14.6	16.7	16.6	24.6*	23.6*	31.8	30.4	34.1	33.5
18:0	1.5	1.4	−	−	−	−	−	−	1.0	1.1
Total saturated	16.6	16.7	18.1	18.1	27.1	26.7	34.1	33.2	36.4	36.0
Monounsaturated fatty acids (MUFA)										
16:1(*n*−9)	1.3	1.3	1.0	1.0	−	−	4.7	4.9	3.5	3.4
16:1(*n*−7)	−	−	1.2	1.2	−	−	1.6	1.6	−	−
18:1(*n*−9)	48.4*	47.4*	50.3	49.7	10.6	10.3	22.7	23.6	35.2	35.4
18:1(*n*−7)	2.2	2.1	3.0**	2.9**	2.0	1.9	2.3	2.4	2.0	1.9
20:1(*n*−9)	−	−	−	−	−	−	3.4	3.2	1.3	1.2
Total MUFA	52.8**	51.7**	55.8*	55.0*	13.8	13.4	35.1	35.9	42.4	42.3
Polyunsaturated fatty acids (PUFA)										
16:2(*n*−7)	−	−	−	−	−	−	−	−	−	−
16:2(*n*−6)	2.6	2.7	2.5	2.6	1.2	1.2	0.9	1.1	1.3	1.3
16:2(*n*−4)	−	−	−	−	−	−	−	−	−	−
16:3(*n*−6)	−	−	−	−	−	−	1.9	1.9	−	−
16:3(*n*−4)	−	−	−	−	−	−	−	−	−	−
16:3(*n*−3)	5.7	6.2	6.1***	6.6***	2.0*	2.1*	−	−	−	−
16:4(*n*−3)	−	−	−	−	11.9	12.9	5.6	5.7	3.1	3.2
16:4(*n*−1)	−	−	−	−	−	−	−	−	−	−
18:2(*n*−6)	10.3	10.3	6.6	6.6	6.3	6.2	6.4	6.4	5.8	5.7
18:3(*n*−6)	−	−	−	−	2.9	2.9	−	−	−	−
18:3(*n*−3)	11.8****	12.2****	10.8*	11.2*	31.4	31.3	7.4	7.6	5.9	6.1
18:4(*n*−3)	−	−	−		1.6	1.6	1.3	1.3	−	−
20:3(*n*−6)	−	−	−	−	−	−	−	−	−	−
20:4(*n*−6)	−	−	−	−	−	−	1.1	1.0	−	−
20:5(*n*−3)	−	−	−	−	−	−	4.1	4.0	1.5	1.5
22:5(*n*−6)	–	−	−	−	−	−	−	−	−	−
22:6(*n*−3)	−	−	−	−	−	−	−	−	−	−
Total *ω*−3 PUFA	17.5***	18.4***	16.9**	17.7**	47.2	48.1	19.4	19.7	12.3	12.9
Total PUFA	30.6***	31.6***	26.1**	27.0**	59.1	59.9	30.8	30.8	21.2	21.7
Total unsatd. FA	83.4	83.3	81.9	81.9	72.9	73.3	65.9	66.8	63.6	64.0
Mean RSD (%)^b^	2.71	3.15	2.40	2.34	10.3	10.9	19.0	18.6	12.7	12.2

Mean values are shown for three biological replicates. Significant differences are shown for the two methods (*t* test)

*P < 0.05; **P < 0.02; ***P < 0.01; ****P < 0.001

^a^ *–* FAMES <1.0 % total (mol%)

^b^Mean relative standard deviation (RSD) determined from FAMES > 1.0 % total (mol%)

**Table 3:** Non-green micro-algal FAME composition (mol%) at stationary phase comparing the direct and Folch methods

Fatty acid	Bacillariophyceae		Eustigmatophyceae		Cryptophyceae		Prymnesiophyceae		Rhodophyceae	
	*T*. *pseudonana*		*N*. *oculata*		*R*. *reticulata*		*I*. *galbana*		*P*. *purpureum*	
	CCAP 1085/12		CCAP 849/1		CCAP 995/2		CCAP 927/1		CCAP 1380/1A	
	Direct	Folch	Direct	Folch	Direct	Folch	Direct	Folch	Direct	Folch
Saturated fatty acids										
14:0	18.0	17.8	5.6	5.7	26.3	26.3	25.6	23.8	−^a^	−
16:0	19.7	19.2	33.5***	32.3***	34.3*	33.7*	15.1	16.2	32.4	31.4
18:0	−	−	1.8	1.7	4.4	4.4	−	−	9.5	9.4
Total saturated	39.4**	38.4**	42.4****	41.1****	65.9	65.3	42.5	41.6	42.8	41.9
Monounsaturated fatty acids (MUFA)										
16:1(*n*−9)	−	−	−	−	−	−	−	−	−	−
16:1(*n*−7)	27.7	27.5	30.3	30.8	1.3	1.3	2.5	2.6	−	−
18:1(*n*−9)	−	−	10.4	9.9	8.0	7.9	18.3	17.3	4.2	4.4
18:1(*n*−7)	−	−	−	−	1.1	1.1	2.1	2.0	−	−
20:1(*n*−9)	−	−	−	−	−	−	−	−	−	−
Total MUFA	29.3	29.2	40.9	40.9	10.6	10.5	23.7	22.6	4.7	5.0
Polyunsaturated fatty acids (PUFA)										
16:2(*n*−7)	2.4	2.5	−	−	−	−	−	−	−	−
16:2(*n*−6)	−	−	−	−	−	−	−	−	−	−
16:2(*n*−4)	3.6	3.6	−	−	−	−	1.3	1.1	−	−
16:3(*n*−6)	−	−	−	−	−	−	−	−	−	−
16:3(*n*−4)	7.7	8.1	−	−	−	−	−	−	−	−
16:3(*n*−3)	−	−	−	−	−	−	−	−	−	−
16:4(*n*−3)	−	−	−	−	−	−	−	−	−	−
16:4(*n*−1)	1.8	2.0	−	−	−	−	−	−	−	−
18:2(*n*−6)	−	−	3.9	3.9	8.7	8.7	3.0	2.8	24.1	24.2
18:3(*n*−6)	−	−	−	−	−	−	−	−	1.1	1.1
18:3(*n*−3)	−	−	−	−	8.5	8.8	6.9	6.7	−	−
18:4(*n*−3)	3.6	3.7	−	−	2.7	2.8	10.8	9.7	−	−
20:3(*n*−6)	−	−	−	−	−	−	−	−	4.6	4.7
20:4(*n*−6)	−	−	2.9	2.9	−	−	−	−	17.6	17.9
20:5(*n*−3)	9.2	9.3	8.4**	9.2**	1.4	1.4	−	−	4.3	4.4
22:5(*n*−6)	−	−	−	−	−	−	2.2	2.3	−	−
22:6(*n*−3)	2.0	1.9	−	−	1.0	1.0	7.4	10.8	−	−
Total *ω*−3 PUFA	15.0	15.3	8.7**	9.7**	13.7	14.3	26.5	28.6	4.4	4.5
Total PUFA	31.3*	32.3*	16.8	18.0	23.5	24.1	33.8	35.8	52.5	53.0
Total unsatd. FA	60.6**	61.6**	57.6****	58.9****	34.1	34.7	57.5	58.4	57.2	58.1
Mean RSD (%)^b^	4.49	4.15	4.33	3.72	3.11	2.32	14.4	5.51	10.3	9.45

Mean values are shown for three biological replicates. Significant differences are shown for the two methods (*t* test)

*P < 0.05; **P < 0.02; ***P < 0.01; ****P < 0.002

^a^– FAMES < 1.0 % total (mol%)

^b^Mean relative standard deviation (RSD) determined from FAMES > 1.0 % total (mol%)

To examine this further, the compositional data was expressed in absolute values (%DW) in Tables 4 and 5. Individual FAME yields were compared between the two methods. In the case of *C*. *ovalis*, where overall extraction efficiency was considerably higher with the direct method (Table 1), individual FAME yields were also significantly higher for this method as expected (Table 4). Of the remaining strains, where there were only minor or non-significant differences in total yields, significant differences in yield for individual FAMES were found in *T*. *pseudonana*, *N*. *oculata* and *R*. *reticulata* (Table 5). In these three strains, virtually all significant differences were confined to PUFAs where it was evident that the direct method was slightly less effective at extracting, or retaining this class of fatty acids. In both methods the anti-oxidant BHT was utilized, but it was noted that BHT levels tended to accumulate in the sample through the Folch procedure judging from the GC-FID traces (Fig. 1). This was attributed to the drying steps in the method concentrating BHT. Despite this observation, adjusting BHT concentrations in the hexane solvent that was present throughout the direct-derivatization procedure (from 0–0.1 % *w*/*v*) resulted in no significant compositional change relative to the original concentration of 0.01 % (*w*/*v*) (data not shown). Therefore, extraction differences rather than lipid peroxidation could be responsible for differences in PUFA yields between the two methods.

**Table 4:** Green micro-algal FAME composition at stationary phase expressed in absolute values (%DW) comparing direct and Folch methods

Fatty acid	Chlorophyceae						Prasinophyceae			
	*C*. *ovalis*		*Chlorella* sp.		*D*. *tertiolecta*		*T*. *chui*		*Tetraselmis* sp.	
	CCAP 211/21A		CCAP 211/75		CCAP 19/27		CCAP 66/21A		CCAP 66/60	
	Direct	Folch	Direct	Folch	Direct	Folch	Direct	Folch	Direct	Folch
Saturated fatty acids										
14:0	−^a^	−	−	−	−	−	0.06	0.07	−	−
16:0	6.74**	4.62**	9.20	9.03	2.16	2.30	1.42	1.32	3.92	3.84
18:0	0.75****	0.50****	−	−	−	−	−	−	0.13	0.13
Total saturated	7.82***	5.35***	10.0	9.85	2.39	2.60	1.52	1.44	4.20	4.14
Monounsaturated fatty acids (MUFA)										
16:1(*n*−9)	0.61*	0.41*	−	−	−	−	0.22	0.23	0.40	0.39
16:1(*n*−7)	−	−	0.65	0.65	−	−	0.07	0.07	−	−
18:1(*n*−9)	24.8***	16.5***	30.5	29.7	1.06	1.15	1.16	1.18	4.47	4.47
18:1(*n*−7)	1.13***	0.75***	1.85	1.72	0.19	0.20	0.13	0.13	0.25	0.24
20:1(*n*−9)	−	−	−	−	−	−	0.18	0.17	0.18	0.17
Total MUFA	27.0***	18.0***	33.7	32.7	1.36	1.47	1.78	1.79	5.34	5.31
Polyunsaturated fatty acids (PUFA)										
16:2(*n*−7)	−	−	−	−	−	−	−	−	−	−
16:2(*n*−6)	1.19***	0.83***	1.37	1.39	0.10	0.12	−	−	0.14	0.15
16:2(*n*−4)	−	−	−	−	−	−	−	−	−	−
16:3(*n*−6)	−	−	−	−	−	−	0.08	0.09	−	−
16:3(*n*−4)	−	−	−	−	−	−	−	−	−	−
16:3(*n*−3)	2.61*	1.92*	3.27	3.47	0.17	0.20	−	−	−	−
16:4(*n*−3)	−	−	−	−	1.00	1.21	0.26	0.27	0.34	0.36
16:4(*n*−1)	−	−	−	−	−	−	−	−	−	−
18:2(*n*−6)	5.25***	3.59***	3.96	3.90	0.62	0.68	0.32	0.31	0.73	0.71
18:3(*n*−6)	−	−	−	−	0.27	0.30	−	−	−	−
18:3(*n*−3)	5.97***	4.19***	6.48	6.58	2.94	3.27	0.39	0.39	0.74	0.76
18:4(*n*−3)	−	−	−	−	0.15	0.16	0.07	0.06	−	−
20:3(*n*−6)	−	−	−	−	−	−	−	−	−	−
20:4(*n*−6)	−	−	−	−	−	−	0.06	0.06	−	−
20:5(*n*−3)	−	−	−	−	−	−	0.23	0.22	0.20	0.20
22:5(*n*−6)	−	−	−	−	−	−	−	−	−	−
22:6(*n*−3)	−	−	−	−	−	−	−	−	−	−
Total *ω*−3 PUFA	8.60**	6.13**	9.77	10.1	4.28	4.86	1.00	1.01	1.50	1.56
Total PUFA	15.1***	10.6***	15.1	15.4	5.42	6.11	1.56	1.55	2.60	2.65
Total unsatd. FA	42.1***	28.6***	48.8	48.1	6.78	7.58	3.34	3.35	7.94	7.96
Mean RSD (%)^b^	7.18	12.0	4.04	4.31	24.5	22.9	33.6	41.4	12.7	12.1

Mean values are shown for three biological replicates. Significant differences are shown for the two methods (*t* test)

*P < 0.05; **P < 0.02; ***P < 0.01; ****P < 0.001

^a^ *–* FAMES < 1.0 % total peak area

^b^Mean relative standard deviation (RSD) determined from FAMES peaks with >1.0 % total peak area

**Table 5:** Non-green micro-algal FAME composition at stationary phase expressed in absolute values (%DW) comparing direct and Folch methods

Fatty acid	Bacillariophyceae		Eustigmatophyceae		Cryptophyceae		Prymnesiophyceae		Rhodophyceae	
	*T*. *pseudonana*		*N*. *oculata*		*R*. *reticulata*		*I*. *galbana*		*P*. *purpureum*	
	CCAP 1085/12		CCAP 849/1		CCAP 995/2		CCAP 927/1		CCAP 1380/1A	
	Direct	Folch	Direct	Folch	Direct	Folch	Direct	Folch	Direct	Folch
Saturated fatty acids										
14:0	2.33	2.44	0.97*	1.18*	10.3	10.5	1.64	1.70	−^a^	−
16:0	2.87	2.95	6.53	7.46	15.1	15.1	1.09	1.32	2.17	2.15
18:0	−	−	0.39	0.43	2.17	2.17	−	−	0.70	0.72
Total saturated	5.46	5.62	8.16	9.39	28.0	28.2	2.86	3.17	2.93	2.94
Monounsaturated fatty acids (MUFA)										
16:1(*n*−9)	−	−	−	−	−	−	−	−	−	−
16:1(*n*−7)	4.00	4.20	5.86	7.06	0.55	0.56	0.18	0.21	−	−
18:1(*n*−9)	−	−	2.22	2.53	3.88	3.93	1.45	1.53	0.31	0.34
18:1(*n*−7)	−	−	−	−	0.53	0.52	0.17	0.18	−	−
20:1(*n*−9)	−	−	−	−	−	−	−	−	−	−
Total MUFA	4.23	4.47	8.11	9.63	5.07	5.14	1.86	1.97	0.35	0.38
Polyunsaturated fatty acids (PUFA)										
16:2(*n*−7)	0.35*	0.37*	−	−	−	−	−	−	−	−
16:2(*n*−6)	−	−	−	−	−	−	−	−	−	−
16:2(*n*−4)	0.51**	0.55**	−	−	−	−	−	−	−	−
16:3(*n*−6)	−	−	−	−	−	−	−	−	−	−
16:3(*n*−4)	1.09	1.22	−	−	−	−	−	−	−	−
16:3(*n*−3)	−	−	−	−	−	−	−	−	−	−
16:4(*n*−3)	−	−	−	−	−	−	−	−	−	−
16:4(*n*−1)	0.26	0.30	−	−	−	−	−	−	−	−
18:2(*n*−6)	−	−	0.84	0.98	4.22	4.29	0.23	0.25	1.77	1.82
18:3(*n*−6)	−	−	−	−	−	−	−	−	0.08	0.08
18:3(*n*−3)	−	−	−	−	4.07***	4.27***	0.54	0.59	−	−
18:4(*n*−3)	0.57	0.61	−	−	1.28****	1.37****	0.84	0.84	−	−
20:3(*n*−6)	−	−	−	−	−	−	−	−	0.36	0.38
20:4(*n*−6)	−	−	0.67*	0.79*	−	−	−	−	1.40	1.45
20:5(*n*−3)	1.57**	1.70**	1.92***	2.51***	0.73*	0.76*	−	−	0.34	0.35
22:5(*n*−6)	−	−	−	−	−	−	0.21	0.23	−	−
22:6(*n*−3)	0.37	0.38	−	−	0.56***	0.60***	0.69	1.11	−	−
Total *ω*−3 PUFA	2.54	2.74	1.99***	2.63***	6.71***	7.10***	2.18	2.66	0.35	0.36
Total PUFA	4.87***	5.32***	3.77**	4.81**	11.5	12.0	2.79	3.31	4.02	4.15
Total unsatd. FA	9.11***	9.79***	11.9*	14.4*	16.5	17.1	4.64	5.29	4.37	4.53
Mean RSD (%)^b^	4.58	4.32	5.58	9.54	3.20	2.24	13.7	32.9	11.9	11.8

Mean values are shown for three biological replicates. Significant differences are shown for the two methods (*t* test)

*P < 0.05; **P < 0.02; ***P < 0.01; ****P < 0.001

^a^ *–* FAMES < 1.0 % total peak area

^b^Mean relative standard deviation (RSD) determined from FAMES peaks with >1.0 % total peak area

## Discussion

A large number of diverse micro-algae species accumulate oil to high levels in the form as TAG (Day et al. 2012; Hu et al. 2008). Mining this diversity of micro-algae requires high-throughput screening approaches to identify those with the most potential. Most surveys of oil content and composition have used solvent extraction methods, of which the Folch-based procedures are the most widely applied (Iverson et al. 2001). Direct-derivatization methods offer a rapid alternative, since extraction and derivatization occur simultaneously (Christie 1993).

A comparative study was carried out to evaluate a direct method and solvent-based extraction on lyophilized material with a representative number of strains and also take into account compositional as well as oil yield effects. Ten species from six different phyla were examined using a scaled-down Folch procedure that was modified to reduce handling time and improve extraction from dry material. This was compared with a direct method that used commercial anhydrous Me-HCl stock (Larson and Graham 2001). This method was one of the simplest in terms of handling steps and use of this catalytic reagent has few reported artefacts (Christie 1993).

Micro-algal TAG contents ranged from 5–60 %DW, with the highest producers being the marine *Chlorella* strains, the cryptophyte *R*. *reticulata* and *N*. *oculata* (Table 1). There was no substantial yield differences between the two methods except for one of the 2 *Chlorella* species (*C*. *ovalis*) where the estimated content was 50 % lower with the modified Folch method (*P* < 0.01). This occurred despite efforts to ensure complete homogenization followed by incubation in solvent. Overall, these data suggest that the direct method is the more reliable for oil content determination in high-throughput screens, whereas the Folch method could potentially underestimate this value in some *Chlorella* species. A similar finding was recently reported for *C*. *vulgaris* showing incomplete extraction with the Soxhlet procedure (Laurens et al. 2012). This is practically important since *Chlorella* and other phenotypically similar chlorococcalean taxa are virtually ubiquitous in terrestrial, freshwater, brackish and marine ecosystem environments and are considered to have much economic potential (Hu et al. 2008). As Folch-based methods continue to be used in lipid analysis of *Chlorella*, this may well be providing underestimates of lipid levels and therefore understating their biotechnological potential.

Extraction difficulties might be connected to specific cell wall characteristics of some *Chlorella* species that appear to impede extraction (Doucha and Lívanský 2008). These factors could be compounded by harvesting the micro-algae at stationary phase. For instance, in *Haematococcus pluvialis*, entry into this phase leads to a strengthening of the cell wall and eventual cyst formation as an adaptation to stress (Aflalo et al. 2007). Factors specific to the *Chlorella* are thought likely to impede uptake of dyes in this genus leading to underestimation of oil content using Nile Red fluorescence (Chen et al. 2009; Sheehan et al. 1998). It is notable that glucosamine polymers such as chitins and chitosan are found in some *Chlorella* species (Kapaun and Reisser 1995; Sun et al. 1999) and genes for chitin remodelling have been identified in the genome of *Chlorella variabilis* (Blanc et al. 2010). Further work is required to establish if there is a relationship between the presence of chitins and obduracy towards extraction in the *Chlorella* genus. How this relates towards the cessation of growth, adaptation towards stress and accumulation of lipids during stationary phase also requires investigation. Considering that the corrosive nature of acid-based derivatization reagents can aid extraction (Browse et al. 1986), it appears that the direct approach for FAME production overcomes these problems in the case of *Chlorella*. From an industrial perspective, acid-catalyzed direct derivatization might be advantageous at the large scale for biodiesel production with these species (Haas and Wagner 2011).

Comparison of relative fatty acid composition between the two methods (Table 2 and 3) revealed low but significant differences (±10 % peak area), with a consistent bias towards lower unsaturation with the direct method. This was observed in the two *Chlorella* strains and *D*. *tertiolecta* (Table 2); *T*. *pseudonana*, *N*. *oculata* and *R*. *reticulata* (Table 3). When the data was expressed in absolute terms (%DW), almost all significant differences were confined to PUFAs where it was evident that the direct method was slightly less effective at extracting, or preserving this class of fatty acids (Tables 4 and 5). Adjusting BHT concentrations in the hexane solvent present during derivatization in the direct method had no effect, suggesting that extraction differences rather than lipid peroxidation might be responsible for differences in PUFA yields (data not shown). In a similar comparison for red blood cells, more saturated fatty acids were extracted with a direct method than with Folch but not MUFAs or PUFAs (Clayton et al. 2012). Conversely, two comparative studies using marine animal material reported improved recovery of PUFAs in direct methods relative to conventional two-step methods, however (Abdulkadir and Tsuchiya 2008; Indarti et al. 2005). This was attributed to greater oxidation associated with the extra handling steps in the latter which was plausible as these studies did not employ antioxidants, as used in the present study. Taken together, it appears that the direct methods are less likely to be impeded in extraction than the Folch-based methods by substrate dependent factors such as cell wall structure but there might be minor reagent based limitations to extraction that discriminate by lipid class or fatty acid composition. The latter could depend on the particular direct method employed and this requires further study.

The selection of future algae production strains for biofuels, food, pharmaceutical, nutraceutical or cosmeceutical purposes necessitates the use of robust efficient lipid analyses suitable for high-throughput screening. In this study, a rapid analytical method (Larson and Graham 2001) and a scaled-down Folch-based procedure (Cook et al. 2000; Folch et al. 1957) were evaluated. Overall, the rapid approach was found to be ideal for high-throughput screening for yield determination, whereas the scaled-down Folch procedure was slightly more accurate in terms of fatty acid composition.
